# Effectiveness of Prophylactic Use of Hepatoprotectants for Tuberculosis Drug-Induced Liver Injury: A Population-Based Cohort Analysis Involving 6,743 Chinese Patients

**DOI:** 10.3389/fphar.2022.813682

**Published:** 2022-04-20

**Authors:** Qin Chen, Airong Hu, Aixia Ma, Feng Jiang, Yue Xiao, Yanfei Chen, Ruijian Huang, Tianchi Yang, Jifang Zhou

**Affiliations:** ^1^ Hwa Mei Hospital, University of Chinese Academy of Sciences, Ningbo, China; ^2^ School of International Pharmaceutical Business, China Pharmaceutical University, Nanjing, China; ^3^ Institute of Tuberculosis Prevention and Control, Ningbo Municipal Center for Disease Control and Prevention, Ningbo, China

**Keywords:** tuberculosis, prophylaxis, high-dimensional propensity score, drug-induced liver injury, pharmacotherapy

## Abstract

**Background:** Tuberculosis drug-induced liver injury (TB-DILI) is a common and potentially severe adverse drug reaction leading to treatment interruption and treatment failure. The real-world preventive effectiveness of hepatoprotective agents for DILI is not well described. The aim of the study was to evaluate the patterns of prophylactic therapies in real-world settings and risks of DILI among adult TB patients without known risk factors for DILI.

**Methods:** This is a population-based retrospective cohort study of patients receiving first-line anti-tuberculosis drugs in the Chinese Center for Disease Control and Prevention (CDC) TB registry linked to the Ningbo Regional Health Care Database (NRHCD) between 2015 and 2020. The primary exposure was any use of chemopreventive agents including silymarin and/or glycyrrhetinic acid during the 30-day period prior to TB diagnosis (index date). The main outcome measure was the occurrence of newly onset DILI following TB treatment. Eligible patients were followed until the earliest of any DILI, treatment discontinuation, death, or end of the study period (30 June 2020). Marginal structural competing risk models and Cox models *via* inverse probability treatment weights using high-dimensional propensity scores were used to estimate subdistribution hazard risks (SHR) and 95% confidence intervals (CIs) for DILI risks, with adjustment for age, sex, TB-related characteristics, and comorbidities.

**Results:** We identified a cohort of 6,743 adult patients with TB (mean age of 47.1 [SD 18.7] years; 65.80% male), of whom 2,886 (42.8%) patients received hepatoprotective agents. A total of 895 DILI events and 111 all-cause death events without DILI were observed over a median follow-up of 367 days post-TB diagnosis. The incidence rates of composite outcomes combining DILI and all-cause mortality were 248.9 and 222.3 per 1,000 person-years in the hepatoprotective agent exposed and unexposed groups (relative hazard ratio 1.35, 95% CI 1.11–1.64), respectively. The incidence rates of DILI were 223.7 and 196.1 per 1,000 person-years in the hepatoprotective agent exposed and unexposed groups (relative hazard ratio 1.38, 95% CI 1.12–1.71), respectively. Patients with any chemopreventive agent use had comparable liver function changes as evidenced by laboratory tests.

**Conclusion:** A non-trivial number of adult patients received chemopreventive agents for TB-DILI. However, prophylactic utilization of hepatoprotective agents was not associated with a reduction in TB-DILI risks.

## Introduction

Drug-induced liver injury (DILI) is a common adverse reaction in anti-tuberculosis (TB) treatment, and potentially severe reactions often lead to treatment interruption and unfavorable outcomes. To prevent DILI, clinicians from TB endemic regions including China routinely prescribe liver protection drugs during anti-tuberculosis treatment, such as silymarin (Marjani et al., 2016; Marjani et al., 2019), phosphatidylcholine, reduced glutathione, milk thistle (Shi et al., 2020), ursodeoxycholic acid (Saito et al., 2016), and glycyrrhizic acid preparations (Saito et al., 2016). Although a number of hepatoprotective agents have been introduced to clinical practice owing to their protective effects on hepatic cells in *in vitro* or *in vivo* experiments, the effectiveness of these drugs in preventing DILI in the real-world settings has not been established yet. While inconclusive hepatoprotective effects have been shown in clinical trials, their uses in clinical settings are controversial (Marjani et al., 2016).

Previous research findings may be limited due to small sample size, unstandardized diagnosis criteria, insufficient follow-up, and lack of comparability. In the absence of head-to-head randomized controlled trials (RCTs), real-world evidence (RWE) studies have the potential to complement RCTs for decision-making by generalizing the findings beyond a specific population (Sherman et al., 2016; Crown, 2019). By examining the associations between exposure of hepatoprotective agents at the time of TB diagnosis and the occurrence of newly diagnosed DILI, RWE could offer important information on causal inference and improve appropriate utilization of hepatoprotective agents (Saito et al., 2016; Oosthuizen et al., 2017; Xu et al., 2017).

The objective of this study was to evaluate the patterns of prophylactic therapies in real-world settings and risks of DILI among adult TB patients without known risk factors for DILI.

## Methods

### Study Cohort Design

We adopted a new user, intention-to-treat retrospective, comparative cohort design approach. Participants who were diagnosed with tuberculosis at designated hospitals in Ningbo between 1 January 2015 and 2 January 2 2020 were initially referred by healthcare institutions throughout the city and then were linked to administrative records from the city’s regional EHR system.

Base cohort entry was defined by the date of the first TB diagnosis (index date). A flowchart of cohort construction is presented in Supplementary Figure S1. With these participants, we selected as the hepatoprotector group those who had newly received at least one prescription of hepatoprotectors as chemoprophylaxis between 30 days and 1 day prior to TB diagnosis. We then limited this group to those with no prior history of hepatoprotector use in the past year, excluding the 1-month initiation window. The schema for subject selection and data collection during the study period is shown in Supplementary Figure S2. Time zero (T_0_, or index date) was defined as the time of TB diagnosis, and we used a 1-year pre-index period as the baseline period. Patients without any use of hepatoprotective agents were included in the control group. To ensure that all participants had comparable covariate ascertainment periods, we only included those who had at least two healthcare encounters on different dates. Both exposed and control groups were further selected to only include participants without abnormal baseline liver function tests and without any known DILI risk factors such as viral hepatitis, history of DILI, pregnancy status, cirrhosis, and other chronic liver disease. We excluded patients with baseline abnormal liver function or patients with a history of viral hepatitis to select patients who were not predisposed to develop DILI. We considered such patients to be at clinical equipoise to receive hepatoprotective agents.

### Data Sources and Data Linkage

The Ningbo Regional Health Database and the Ningbo Community CDC TB registry were used in the present study (Zhang et al., 2018). Outpatient and inpatient encounter domains were used to collect International Classification of Diseases, tenth version (ICD-10) diagnosis codes. The inpatient and outpatient pharmacy domains were used to collect prescription data and provider data. Inpatient and outpatient laboratory results were captured from the laboratory result domain. Vital measurements and medical history were collected from the vital sign and health chart domains. Demographic information, including date and cause of death, was collected in the vital status databases linked to the death registries of the Ningbo Public Security Bureau. The regional healthcare database contains information on anthropometric variables such as body weight and height, BMI, and lifestyle variables such as smoking and alcohol use status. We linked the CDC TB registry and regional health database to generate a cohort including patient information both before and after TB diagnosis.

TB-related characteristics were captured from the CDC TB registry; Information on TB diagnosis date, retreatment regimen, and drug-susceptibility test (DST) as well as treatment outcomes were extracted.

### Outcome

The outcome of DILI was defined using the updated CSH (Chinese Society of Hepatology, Chinese Medical Association) DILI consensus (Supplemental Table S1) (Yu et al., 2017). Patients meeting the study inclusion criteria were followed until the earliest of the following events: TB treatment discontinuation or completion, death, occurrence of DILI, or end of the study period (30 June 2020).

The outcome of DILI was defined as the time until the first occurrence of DILI, or all-cause mortality. We also estimated the trajectory of ALT, ALP, and total bilirubin values during follow-up.

### Exposure

Prescriptions of any hepatoprotectors were identified from inpatient and outpatient pharmacy records. Both parenteral and oral hepatoprotective agents were analyzed. Treatment of liver protectant agent status was considered 0 and 1 before and following initiation, respectively.

Classes of hepatoprotectors include: silymarin, glycyrrhetinic acid, and others.

### Covariates

Predictors of hepatoprotector prescription were selected as predefined covariates and ascertained in the year before T_0_. Predefined covariates included age, sex, insurance type, smoking and alcohol consumption status, baseline ALT, calendar year of Tb diagnosis, extrapulmonary TB status, TB retreatment status, ALP, and total bilirubin.

We also included medical conditions that may affect the choice of liver-protective agents, such as COPD, diabetes, hypertension, acute kidney injury, etc. The comorbidity burdens were assessed using the Charlson comorbidity index.

To mitigate potential biases that were not adjusted for the predefined covariates, high-dimensional propensity scores were estimated using the EHR diagnosis and pharmacy information within the 1-year pre-index baseline period (Schneeweiss et al., 2009). Inpatient and outpatient ICD-10 codes, in addition to pharmacy records, were used by the empirical variable selection algorithm to find the 500 most frequently occurring items from each of the four domains. Each item (diagnosis code or drug name from both inpatient and outpatient settings) was categorized into three binary variables: ever, sporadic, and frequently occurring, which generated a total of 500 × 4 × 3 = 6,000 variables. The top 200 variables with the largest risk ratios in their association with assignment to a hepatoprotector user or control group were selected. We conducted high-dimensional variable selection independently in the overall cohort and within each subgroup for subgroup analyses (Schneeweiss et al., 2009).

### Ethical Approval

The studies involving human participants were reviewed and approved by the Ningbo CDC Institutional Review Board (IRB) (Approval No. 202201), as well as Hwa Mei Hospital IRB (Approval No. YJ-NBEY-KY-2021-190-01). The Ethics Committees waived the requirement of written informed consent for participation.

### Statistical Analysis

Characteristics of the overall cohort and within the hepatoprotector user and control groups were presented as mean and SD, or number and percentage, as appropriate. Missing data was handled using multiple imputation analysis, with resulting estimates from each of these pooled sets (Donders et al., 2006; Li et al., 2015). Time until DILI and all-cause mortality following the index date was assessed using the Kaplan-Meier method, and log-rank tests were used to compare the equality of survivor functions.

We used the inverse probabilistic treatment weighting (IPTW) approach to adjust for confounding. The probability of new hepatoprotector initiation on or before the TB treatment start date, in the form of a high-dimensional propensity score, was estimated from a multivariable logistic regression. The exposed and control groups were weighted based on the probability of initiating hepatoprotectors during the drug initiation window, with 1/probability of initiating hepatoprotectors, or 1/(1- probability of initiating hepatoprotectors), respectively. In the weighted pseudocohort, the subjects with treatment weighting above 99th percentile were trimmed from the cohort. We presented the characteristics in the weighted and unweighted cohorts, as well as the absolute standardized difference between groups to demonstrate the reduction in baseline characteristics. Weighted survival probabilities in the form of Kaplan-Meier curves are also presented. Adjusted hazard ratios (HRs) and 95% confidence intervals (CIs) for the association between hepatoprotector exposure and outcomes were estimated using high-dimensional propensity score-weighted multivariable Cox proportional hazards models.

We applied a weighted linear mixed model to estimate the ALT, ALP, and total bilirubin value trajectories in the weighted pseudocohort. A cubic spline of time was used to capture potential non-linear trajectories during the follow-up period, where knots were placed at 0, 90, 180, and 365 days after the index date. The differences in laboratory results between treatment arms were estimated from the interaction between treatment group status and the splined time. Baseline values as well as those at 90, 180, and 365 days were plotted. The differences between the two trajectories, where the control group served as the reference, represented the changes associated with hepatoprotective agents at each time point, after accounting for potential confounding factors.

Analyses were conducted using SAS 9.4 statistical software and R 4.0.3 statistical software. A two-side *p* value < 0.05 was considered statistically significant.

## Results

### Sample Description

During the study period, 12,087 patients with TB were identified from linked databases. We excluded 5,344 patients for the following reasons: In 406 patients, the initial TB diagnosis changed; at baseline, 1,558 lacked sufficient clinical information; 14 lacked any records of clinical encounters after TB diagnosis; 140 were non-adults; 151 were pregnant; 1,668 had abnormal baseline liver function; 562 had pre-existing liver diseases, 324 had history of pregnancy during TB treatment and 521 had prior exposure to any hepatoprotective agents. The final cohort included 6,743 adult patients with TB (mean age of 47.1 [SD 18.7] years; 65.80% male), of whom 2,886 (42.8%) patients received hepatoprotective agents. The majority of patients in the exposed group received silymarin (46.5%) and glycyrrhizin (39.0%).

After a median follow-up of 367 days post-TB diagnosis, 895 DILI events and 111 all-cause death events without DILI were identified among eligible patients.

### Baseline Characteristics

As shown in Table 1, patients in the exposed group were younger than those in the control group. Additionally, they were more likely to be of Han ethnicity and had extrapulmonary TB. In terms of pre-existing comorbidities, patients who received hepatoprotective agents were more likely to have myocardial infarction (0.3% vs. 0.1%, *p* = 0.040), chronic heart failure (0.4% vs. 0.3%, *p* = 0.007), COPD (9.0% vs. 8.6%, *p* = 0.012), type 2 diabetes (8.2% vs. 7.2%, *p* = 0.036), and malignant disease (5.6% vs. 5.0%, *p* = 0.026).

**TABLE 1:** Demographic and clinical characteristics of the cohort before and after high-dimensional propensity score weighting.

Characteristic^a^	Control group	Exposed group	STD	Control group	Exposed group	STD
*N*	(*N* = 3,857)	(*N* = 2,886)		(*N* = 6,766.98	(*N* = 6,690.33)	
Age at TB diagnosis [mean (SD)]	47.23 (18.70)	46.94 (18.64)	0.016	47.27 (18.74)	46.88 (18.52)	0.021
Sex (%)						
Male	2,546 (66.0)	1893 (65.6)	0.009	4,480.6 (66.2)	4,421.8 (66.1)	0.003
Female	1,311 (34.0)	993 (34.4)		2,286.4 (33.8)	2,268.6 (33.9)	
Ethnicity (%)						
Han	3,767 (97.7)	2,835 (98.3)	0.042	6,616.7 (97.8)	6,572.1 (98.3)	0.035
Non-Han	88 (2.3)	49 (1.7)		147.5 (2.2)	113.4 (1.7)	
Retreatment status						
Initial treatment	3,644 (94.5)	2,738 (94.9)	0.018	6,397.2 (94.5)	6,347.6 (94.9)	0.015
Retreatment	213 (5.5)	148 (5.1)		369.8 (5.5)	342.7 (5.1)	
TB types						
Extrapulmonary TB	278 (7.2)	236 (8.2)	0.036	492.6 (7.3)	547.6 (8.2)	0.034
Pulmonary TB	3,579 (92.8)	2,650 (91.8)		6,274.4 (92.7)	6,142.7 (91.8)	
Marital status (%)						
Married	1,988 (69.5)	1,534 (67.2)	0.054	3,408.6 (68.6)	3,560.4 (67.8)	0.033
Single	504 (17.6)	416 (18.2)		904.3 (18.2)	944.1 (18.0)	
Other	370 (12.9)	332 (14.5)		653.3 (13.2)	750.0 (14.3)	
Myocardial infarction (%)						
Yes	4 (0.1)	8 (0.3)	0.040	7.1 (0.1)	25.0 (0.4)	0.055
No	3,853 (99.9)	2,878 (99.7)		6,759.9 (99.9)	6,665.3 (99.6)	
Chronic heart failure (%)						
Yes	13 (0.3)	11 (0.4)	0.007	22.1 (0.3)	27.8 (0.4)	0.015
No	3,844 (99.7)	2,875 (99.6)		6,744.9 (99.7)	6,662.6 (99.6)	
Cardiovascular disease (%)						
Yes	55 (1.4)	30 (1.0)	0.035	78.6 (1.2)	69.0 (1.0)	0.012
No	3,802 (98.6)	2,856 (99.0)		6,688.4 (98.8)	6,621.3 (99.0)	
COPD (%)						
Yes	333 (8.6)	259 (9.0)	0.012	566.5 (8.4)	538.7 (8.1)	0.012
No	3,524 (91.4)	2,627 (91.0)		6,200.5 (91.6)	6,151.6 (91.9)	
Type 2 diabetes mellitus (%)						
Yes	278 (7.2)	236 (8.2)	0.036	498.0 (7.4)	496.2 (7.4)	0.002
No	3,579 (92.8)	2,650 (91.8)		6,269.0 (92.6)	6,194.1 (92.6)	
Chronic kidney disease (%)						
Yes	12 (0.3)	10 (0.3)	0.006	20.4 (0.3)	28.6 (0.4)	0.021
No	3,845 (99.7)	2,876 (99.7)		6,746.5 (99.7)	6,661.7 (99.6)	
Malignant disease (%)						
Yes	194 (5.0)	162 (5.6)	0.026	322.6 (4.8)	325.5 (4.9)	0.005
No	3,663 (95.0)	2,724 (94.4)		6,444.4 (95.2)	6,364.8 (95.1)	
HIV infection (%)						
Yes	6 (0.2)	5 (0.2)	0.004	13.1 (0.2)	12.1 (0.2)	0.003
No	3,851 (99.8)	2,881 (99.8)		6,753.9 (99.8)	6,678.3 (99.8)	
Smoking (%)						
Former smoker	129 (3.3)	97 (3.4)	0.019	227.1 (3.4)	225.2 (3.4)	0.016
No smoker	1,318 (34.2)	987 (34.2)		2,243.3 (33.2)	2,236.8 (33.4)	
Smoker	334 (8.7)	238 (8.2)		571.8 (8.4)	546.2 (8.2)	
Missing	2,076 (53.8)	1,564 (54.2)		3,724.8 (55.0)	3,682.1 (55.0)	
Alcohol (%)						
Drinker	140 (3.6)	99 (3.4)	0.011	239.5 (3.5)	243.2 (3.6)	0.011
Never	1,609 (41.7)	1,187 (41.1)		2,747.0 (40.6)	2,681.0 (40.1)	
Missing	2,108 (54.7)	1,600 (55.4)		3,780.5 (55.9)	3,766.1 (56.3)	
BMI [mean (SD)]	21.72 (5.61)	22.34 (8.58)	0.085	21.95 (6.79)	22.31 (8.02)	0.049

a Values are numbers (percentages) unless stated otherwise.

TB, tuberculosis; SD, standard deviation; IQR, interquartile range; STD, standardized difference; HIV, human immunodeficiency virus.

Weighting for observable selection bias led to excellent balance for the majority of the covariates, with a standardized mean difference (STD) generally lower than 0.1.

### Frequency of Incident Preventive Hepatoprotective Agent Use

Overall, 1,342 (46.5%) participants used silymarin as a prophylactic DILI agent, followed by glycyrrhizin (1,125, 39.0%). Other hepatoprotective agents included Artemisiae, chlorophyllin, phosphatidylcholine, Schisandra, and cell factors. Their respective DILI risks are presented in Table 2. Patients exposed to silymarin (225.5 per 1,000 person-years, 95% CI 194.0–257.0) and glycyrrhizin (232.7 per 1,000 person-years, 95% CI 197.2–268.2) had higher incidence rates of DILI as compared to those in the control group (196.1 per 1,000 person-years, 95% CI 178.6–213.7).

**TABLE 2:** Frequency of new initiation of preventive hepatoprotective agents in users.

	No. of patients	No. of DILI events	Follow-up duration (person-years)	DILI event incidence per 1,000 person-years (95%CI)
Control group	3,857	479	2,442.3	196.1 (178.6–213.7)
Silymarin	1,342	197	873.7	225.5 (194.0–257.0)
Glycyrrhizin	1,125	165	709.2	232.7 (197.2–268.2)
Miscellaneous^a^				
Artemisiae	133	18	84.4	213.3 (114.7–311.8)
Chlorophyllin	2	0	1.9	NA
Phosphatidylcholine	58	5	42.4	117.9 (14.6–221.3)
Schisandra	225	31	146.5	211.6 (137.1–286.1)
Cell factor	1	0	1.0	NA

a Miscellaneous liver-protective agents include Artemisiae, chlorophyllin, phosphatidylcholine, Schisandra, and cell factor.

DILI, drug-induced liver injury; NA, not applicable.

### Incidence Rate of Drug-Induced Liver Injury and All-Cause Mortality

Of the 6,743 eligible patients, over 4,302 person-years of follow-up, a total of 895 patients (13.3%) developed DILI and 111 patients (1.6%) died. The incidence rates of composite outcomes were 248.9 per 1,000 person-years in the exposed group, compared to 222.3 per 1,000 person-years in the reference group. The incidence rate difference was 26.6 (95% CI, −2.81–55.97) per 1,000 person-years. No statistical differences were observed between exposed and reference groups on either DILI or all-cause mortality, with incidence rate differences of 27.5 (95% CI −0.2–55.3) and -0.9 (95% CI −10.6, 8.7) per 1,000 person-years (Table 3). Subgroups analyses on respective types of liver-protective agents were shown in Supplementary Table S2. In subgroup analyses, we found that the overall rates of DILI and all-cause deaths were higher in the glycyrrhetinic acid exposed group (Adjusted HR = 1.73, 95% CI 1.31–2.28), while no statistically significant difference was observed between the silymarin exposed and reference groups (Adjusted HR = 0.90, 95% CI 0.71–1.14).

**TABLE 3:** Crude and adjusted hazard ratios for the association between the use of liver-protective agents and the risk of the study outcomes.

Outcome	No. of patients	No. of events	Person years	Incidence rate per 1,000 person-years	Event difference per 1,000 person-years (95% CI)	Crude hazard ratio (95%CI)	Adjusted hazard ratio (95% CI)
Overall^a^							
User	2,886	463	1,860.1	248.9	26.6 (−2.8, 56.0)	1.35 (1.17–1.56)	1.35 (1.11–1.64)
Non-user	3,857	543	2,442.3	222.3			
DILI^a^							
User	2,886	416	1,860.1	223.7	27.5 (−0.2,55.3)	1.34 (1.15–1.56)	1.38 (1.12–1.71)
Non-user	3,857	479	2,442.3	196.1			
All-cause mortality^b^							
User	2,886	47	1,860.1	25.3	−0.9 (−10.6, 8.7)	1.44 (0.96–2.17)	1.72 (0.96–3.08)
Non-user	3,857	64	2,442.3	26.2			

a The models for overall DILI-free survival and DILI events were adjusted for age, sex, TB diagnosis year, high-dimensional propensity score, baseline AST, ALP, and total bilirubin results, and comorbidity burdens in the year before TB diagnosis.

b The model for all-cause mortality was adjusted for age, sex, TB diagnosis year, retreatment status, high-dimensional propensity score, and comorbidity burdens in the year before TB diagnosis.

**FIGURE 1:**
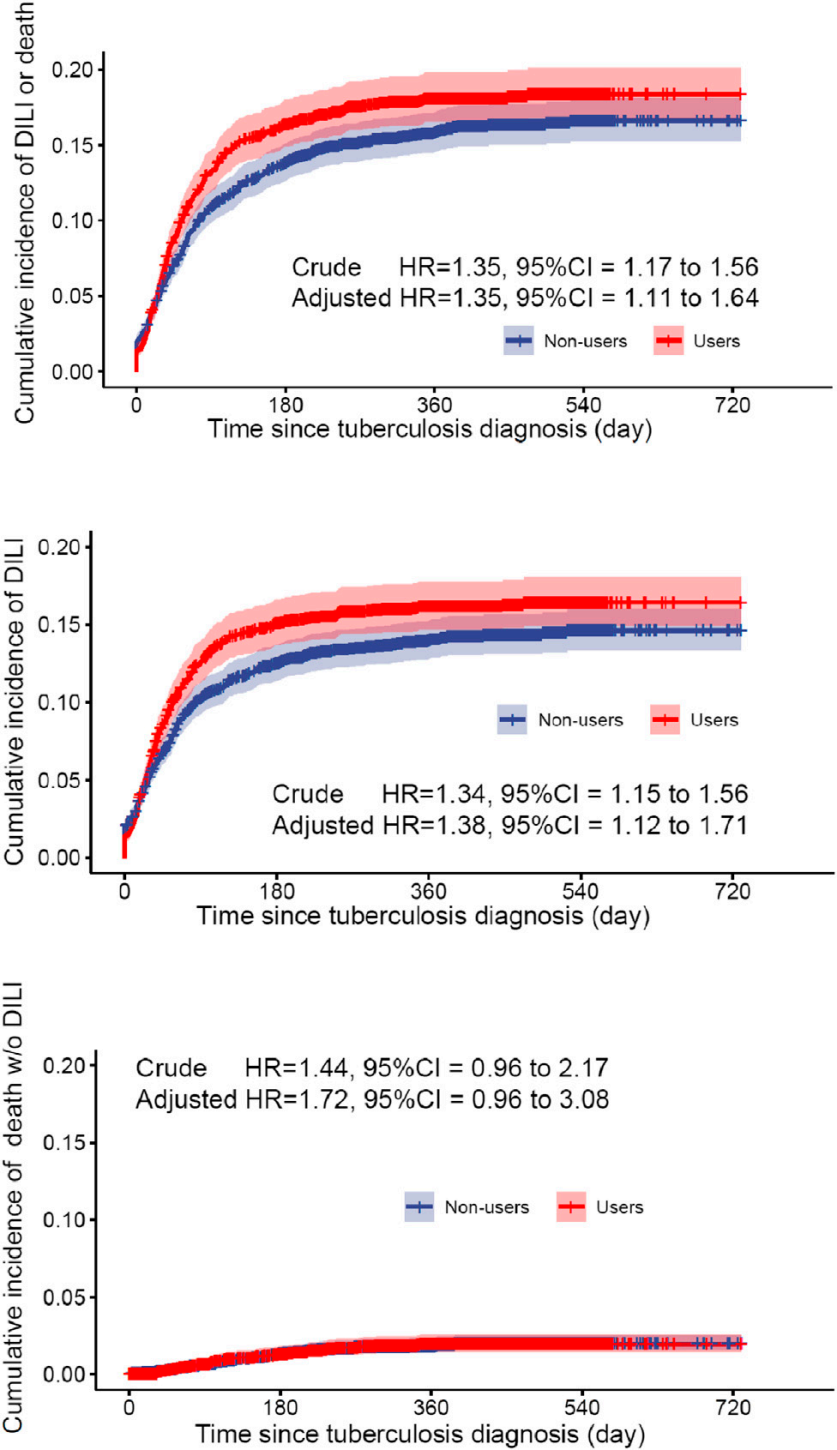
Survival probability for DILI or all-cause mortality in the hepatoprotective agent user group and the control group in the weighted cohort.

The cumulative incidence of DILI and all-cause mortality between exposed and reference groups are shown in Figure 1. Results of univariate Cox regression analyses demonstrated that prophylactic use of hepatoprotective agents was associated with statistically significant elevated risks for DILI and all-cause death (HR = 1.35, 95% CI 1.17–1.56). After accounting for age, sex, TB diagnosis year, high-dimensional propensity score, baseline AST, ALP, and total bilirubin results, and comorbidity burdens in the year before TB diagnosis, the propensity-score weighted multivariable Cox regression model showed that use of liver-protective agents was associated with 35% increases in composite outcome (HR = 1.35, 95% CI 1.11–1.64). On the other hand, cumulative incidence rates of all-cause mortality were comparable between exposed and unexposed groups (adjusted HR = 1.72, 95% CI 0.96–3.08) (Table 3).

### ALT, ALP, and Total Bilirubin Trajectories During Follow-Up

**FIGURE 2:**
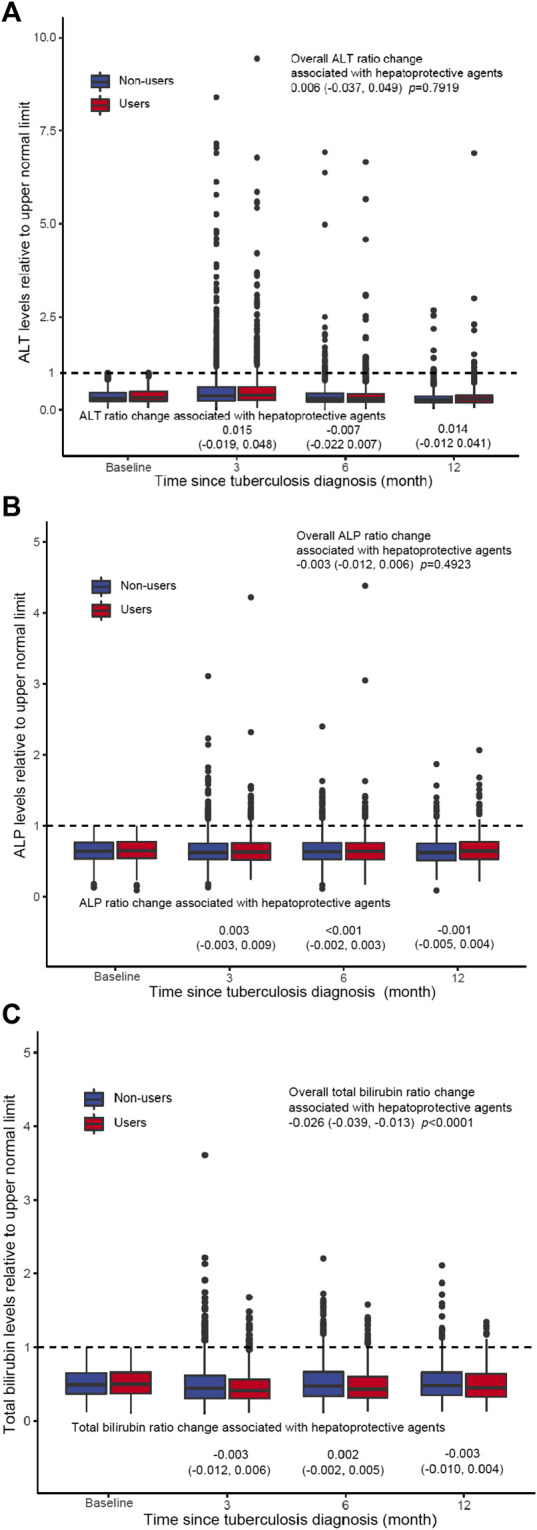
ALT **(A)**, ALP **(B)**, and total bilirubin **(C)** trajectories during follow-up. Estimated laboratory values and 95% CI at baseline for 90, 180 days, and 1 year were plotted for the hepatoprotective agent users and non-users. Laboratory test value changes associated with liver-protective agent utilization at each time point represent the difference between the two trajectories in which the non-user (unexposed) group served as a reference.

Estimated trajectories of ALT, ALP and total bilirubin values relative to the upper limit of normal (ULN) in the exposed and control groups showed similar trends (Figure 2). Both groups experienced elevation in aminotransferases enzyme and total bilirubin after TB treatment initiation, the exposed group was associated with minimal but statistical significant reduction in total bilirubin values than the control group (−0.026 95% CI −0.039, −0.013, *p* < 0.0001). Compared with the control group, use of hepatoprotective agents was not associated with changes in ALT and ALP values at 3, 6, and 12 months after tuberculosis diagnosis.

## Discussion

In this real-world study of patients with tuberculosis, the incident use of hepatoprotective agents versus the reference group was associated with increases in DILI events. The association persisted after accounting for baseline characteristics using hdPS weighting. Additionally, the subgroup analyses indicated that different types of liver-protective agents had varying effects on the incidence of DILI and all-cause mortality. Our results suggest that all hepatoprotective agents, excluding silymarin, were associated with increased DILI risk. This is consistent with other randomized clinical trials that have shown conflicting findings on the prophylactic use of liver-protective agents (Chen et al., 2015; Tao et al., 2019).

Hepatoprotective therapy is the application of drugs to reduce liver cell and tissue damage and promote repair and regeneration of damaged liver cells, thereby improving liver biochemical indicators and restoring liver function. It is worth noting that one of the functions of the liver is to decompose and transform the various components of drugs. Long-term use of hepatoprotective drugs is likely to increase the burden on the liver invisibly. At the same time, some hepatoprotective drugs accumulate in the body for a long time, which can also cause damage to the liver. As far as we know, the effectiveness of hepatoprotective drugs has long been under debate (Saito et al., 2016; Zhang et al., 2016; Xu et al., 2017). Baniasadi et al. (2010) assessed the impacts of hepatoprotective agents on 60 older TB patients, and they found that N-acetylcysteine (NAC) may have protected patients from developing DILI following TB treatment. However, this study suffered from limited sample size, short follow-up duration, and heterogeneous patient distribution. Some meta-analyses of earlier published articles concluded that hepatoprotective drugs may prevent drug-induced liver injury in patients receiving anti-TB drugs, especially after 4 weeks of hepatoprotective medication (Xu et al., 2017). However, some RCT studies and large population cohort studies have reached the opposite conclusion; that is, prophylactic use of hepatoprotective agents not only has no preventive effect on drug-induced liver injury but may even increase the risk of its occurrence (Marjani et al., 2016; Marjani et al., 2019; Tao et al., 2019). Our research findings are consistent with the latter. Considering the wide variety of liver-protecting drugs and different mechanisms of action, we also explored the effects of different types of drugs on preventive liver-protecting treatments. However, it was found that, except for silymarin, all others were associated with an increased risk of liver damage. In China, the expert recommendations for the diagnosis and treatment of drug-induced liver injury caused by anti-tuberculosis drugs in 2013 and 2017 clearly pointed out that there is still insufficient evidence to support routine preventive hepatoprotective treatment for patients without high-risk factors (Yu et al., 2017). However, physicians still routinely prescribe various hepatoprotective drugs during anti-tuberculosis treatment on the grounds of preventing liver damage, such as glycyrrhizic acid preparations, reduced glutathione, bicyclic alcohol, silymarin preparations, troponin, essential phospholipids, and glucurolactones (Wu et al., 2015). Some studies believe that this phenomenon is the result of the supplier’s pursuit of financial benefits under the current medical benefit-driven mechanism (Ma et al., 2019). Considering that under the current National Tuberculosis Control Program in China, liver protection drugs are not provided free of charge. Over-prescription of these medications to patients may greatly increase the economic burden, thereby affecting the process of tuberculosis prevention and control (Li et al., 2012). Therefore, curbing the abuse of non-medical factors in liver-protecting drugs is not only a medical problem, but also a public health problem. Policies aimed at combating hepatoprotective agent misuse’ problem require a systemic approach involving medical community, government and drug regulatory agency.

This study has several strengths, including sampling from a large representative cohort of Chinese patients with active TB during a contemporary treatment period (2015–2020) with detailed information on mediation use from both inpatient settings and outpatient pharmacy dispensing records. We employed laboratory findings to further validate the occurrence of DILI following TB treatment. We excluded patients with known risk factors for DILI as well as those with abnormal liver function test findings prior to TB treatment. We built our new-user cohort consisting of patients newly initiating prophylactic treatment. Furthermore, we measured and adjusted for potential selection bias using a high-dimensional propensity score as a possible indicator for initiating liver-protective agents.

There are a number of limitations to our study which should be considered when interpreting our findings. We lacked important TB-related medication use information as the drugs were dispensed to patients at no out-of-pocket costs and not routinely collected in the EHR, which limited our ability to control TB treatment adherence and duration. Despite the use of the hdPS and IPTW design, there might have been residual unmeasured confounding factors. Moreover, we relied on diagnosis codes extracted from administrative data to identify clinical events of interest, and therefore our findings are subjected to misclassification bias and inaccuracies. Due to high rates of missingness on lifestyle characteristics such as smoking status and alcoholism, these variables were not included in the model, which could introduce biases from unmeasured confounding factors. Additionally, we were unable to ascertain the types or duration of anti-TB medications in eligible patients under treatment. This is because anti-TB drugs were dispensed free of charge through a public health facility network rather than pharmacies in China. Last, we assumed that patients are taking drugs diligently and without omission; however, our drug exposure reflects dispensing and not ingestion, which could lead to underestimation of treatment effects.

In conclusion, we observed a positive association between prophylactic use of hepatoprotective agents and the risk of DILI and all-cause mortality. Our findings warrant additional investigation well designed to include prospective clinical trials to elucidate comparative effectiveness of liver-protective agents. Such results are essential to inform decision-making in patients without DILI risks at TB treatment initiation.

## Data Availability

The raw data supporting the conclusion of this article will be made available by the authors, without undue reservation.
