# Supplementary material for: Effectiveness of Prophylactic Use of Hepatoprotectants for Tuberculosis Drug-Induced Liver Injury: A Population-Based Cohort Analysis Involving 6,743 Chinese Patients
Source: Front Pharmacol. 2022 Apr 20;13:813682. doi: 10.3389/fphar.2022.813682 (PMC9065346; doi:10.3389/fphar.2022.813682)

**Supplemental Tables and Figures**

**Supplemental Table 1:** Definition and criteria for drug-induced liver injury

**Supplemental Table 2:** Subgroup analyses on different types of medication exposures and outcomes

**Supplemental Figure 1**: Patient flow diagram

**Supplemental Figure 2**: Schematic overview of study design

**Supplemental Table 1:** Definition and criteria for drug-induced liver injury

| **Grade level** | **Definition** | **Description** |
| --- | --- | --- |
| 0 | No liver injury | Patients tolerate drug treatment and have no hepatotoxic reactions. |
| 1 | Mild liver injury | Elevations in serum ALT and/or ALP levels, TBil <2.5 ULN (2.5 mg/dL or 42.75 μmol/L), INR <1.5. Most patients show adaptability to the liver injury. Patients may present with or without symptoms such as fatigue, asthenia, nausea, anorexia, right upper abdominal pain, jaundice, pruritus, rashes, or weight loss |
| 2 | Moderate liver injury | Elevated serum ALT and/or ALP, with TBil ≥2.5 ULN or INR ≥1.5. The above mentioned symptoms may become aggravated. |
| 3 | Severe liver injury | Elevated serum ALT and/or ALP, TBil ≥5 ULN (5 mg/dL or 85.5 μmol/L) with or without INR ≥1.5. The symptoms are further aggravated, which indicates the need of hospitalization or delayed hospital stay, but there is no evidence of hepatic encephalopathy. |
| 4 | ALF | Evidence of coagulation abnormality indicated by INR ≥1.5 or PTA <40%, signs of hepatic encephalopathy, and TBil ≥10 ULN (10 mg/dL or 171 μmol/L) or daily elevation ≥1.0 mg/dL (17.1 μmol/L) in 26 weeks after the DILI onset. Patients may have ascites and DILI-related dysfunction of other organs. If there is evidence of underlying chronic liver diseases, especially liver cirrhosis, the diagnosis of ACLF is established. |
| 5 | Lethal | Death due to DILI, or need to receive liver transplantation for survival. |

Abbreviation: ALT, Alanine transaminase; ALP, Alkaline phosphatase; TBil, Total bilirubin; INR, International normalized ratio; ALF, Acute liver failure; ULN, upper limit of normal; PTA, Plasma thromboplastin antecedent; DILI, drug-induced liver injury; ACLF, Acute-on-chronic liver failure,

**Supplemental Table 2:** Subgroup analyses on different types of medication exposures and outcomes

|  | **No of patients** | **No of events** | **Person years** | **Incidence rate per 1000 person-years** | **Event difference per 1000 person-years (95% CI)** | **Crude hazard ratio (95%CI)** | **Adjusted hazard ratio (95%CI)** |
| --- | --- | --- | --- | --- | --- | --- | --- |
| **Overall*** | | | | | | | |
| Silymarin exposed group | 1342 | 223 | 873.7 | 255.3 | 32.9 (-5.5,71.3) | 0.76 (0.63-0.91) | 0.90 (0.71-1.14) |
| Non-users | 3857 | 543 | 2442.3 | 222.3 |  |  |  |
| **DILI**** | | | | | | | |
| Silymarin exposed group | 1342 | 197 | 873.7 | 225.5 | 29.4 (-6.7,65.4) | 0.76 (0.63-0.92) | 0.94 (0.73-1.20) |
| Non-users | 3857 | 479 | 2442.3 | 196.1 |  |  |  |
| **All-cause mortality**** | | | | | | | |
| Silymarin exposed group | 1342 | 26 | 873.7 | 29.8 | 3.6 (-9.6, 16.7) | 0.75 (0.44-1.28) | 1.45 (0.58-3.67) |
| Non-users | 3857 | 64 | 2442.3 | 26.2 |  |  |  |

* The models for overall DILI-free survival and DILI events were adjusted for age, sex, TB diagnosis year, high-dimensional propensity score, baseline AST, ALP and total bilirubin results, and comorbidity burdens in the year before TB diagnosis.

**The model for all-cause mortality was adjusted for age, sex, TB diagnosis year, retreatment status, high-dimensional propensity score, and comorbidity burdens in the year before TB diagnosis.

|  | **No of patients** | **No of events** | **Person years** | **Incidence rate per 1000 person-years** | **Event difference per 1000 person-years (95% CI)** | **Crude hazard ratio (95%CI)** | **Adjusted hazard ratio (95%CI)** |
| --- | --- | --- | --- | --- | --- | --- | --- |
| **Overall*** | | | | | | | |
| Glycyrrhetinic acid exposed group | 1125 | 177 | 709.2 | 249.6 | 27.3 (-14.0, 68.5) | 1.20 (1.01-1.43) | 1.73 (1.31-2.28) |
| Non-users | 3857 | 543 | 2442.3 | 222.3 |  |  |  |
| **DILI**** | | | | | | | |
| Glycyrrhetinic acid exposed group | 1125 | 165 | 709.2 | 232.7 | 36.5 (-3.1, 76.1) | 1.33 (1.11-1.60) | 1.96 (1.45-2.65) |
| Non-users | 3857 | 479 | 2442.3 | 196.1 |  |  |  |
| **All-cause mortality**** | | | | | | | |
| Glycyrrhetinic acid exposed group | 1125 | 12 | 709.2 | 16.9 | -9.3 (-20.8, 2.2) | 0.34 (0.17-0.68) | 0.22 (0.04-1.35) |
| Non-users | 3857 | 64 | 2442.3 | 26.2 |  |  |  |

* The models for overall DILI-free survival and DILI events were adjusted for age, sex, TB diagnosis year, high-dimensional propensity score, baseline AST, ALP and total bilirubin results, and comorbidity burdens in the year before TB diagnosis.

**The model for all-cause mortality was adjusted for age, sex, TB diagnosis year, retreatment status, high-dimensional propensity score, and comorbidity burdens in the year before TB diagnosis.

|  | **No of patients** | **No of events** | **Person years** | **Incidence rate per 1000 person-years** | **Event difference per 1000 person-years (95% CI)** | **Crude hazard ratio (95%CI)** | **Adjusted hazard ratio (95%CI)** |
| --- | --- | --- | --- | --- | --- | --- | --- |
| **Overall*** | | | | | | | |
| Other hepatoprotective users ** | 419 | 63 | 277.2 | 227.2 | 4.9 (-54.2, 64.1) | 0.85 (0.70-1.04) | 1.84 (1.32-2.56) |
| Non-users | 3857 | 543 | 2442.3 | 222.3 |  |  |  |
| **DILI***** | | | | | | | |
| Other hepatoprotective users** | 419 | 54 | 277.24 | 194.8 | -1.4 (-56.2, 53.5) | 0.70 (0.55-0.87) | 2.03 (1.40-2.95) |
| Non-users | 3857 | 479 | 2442.3 | 196.1 |  |  |  |
| **All-cause mortality***** | | | | | | | |
| Other hepatoprotective users** | 419 | 9 | 277.2 | 32.5 | 6.3 (-15.9, 28.4) | 1.92 (1.21-3.06) | 1.30 (0.41-4.18) |
| Non-users | 3857 | 64 | 2442.3 | 26.2 |  |  |  |

* The models for overall DILI-free survival and DILI events were adjusted for age, sex, TB diagnosis year, high-dimensional propensity score, baseline AST, ALP and total bilirubin results, and comorbidity burdens in the year before TB diagnosis.

** New hepatoprotective agent users other than glycyrrhetinic acid and silymarin.

***The model for all-cause mortality was adjusted for age, sex, TB diagnosis year, retreatment status, high-dimensional propensity score, and comorbidity burdens in the year before TB diagnosis.

**Supplemental Figure 1**: Patient flow diagram


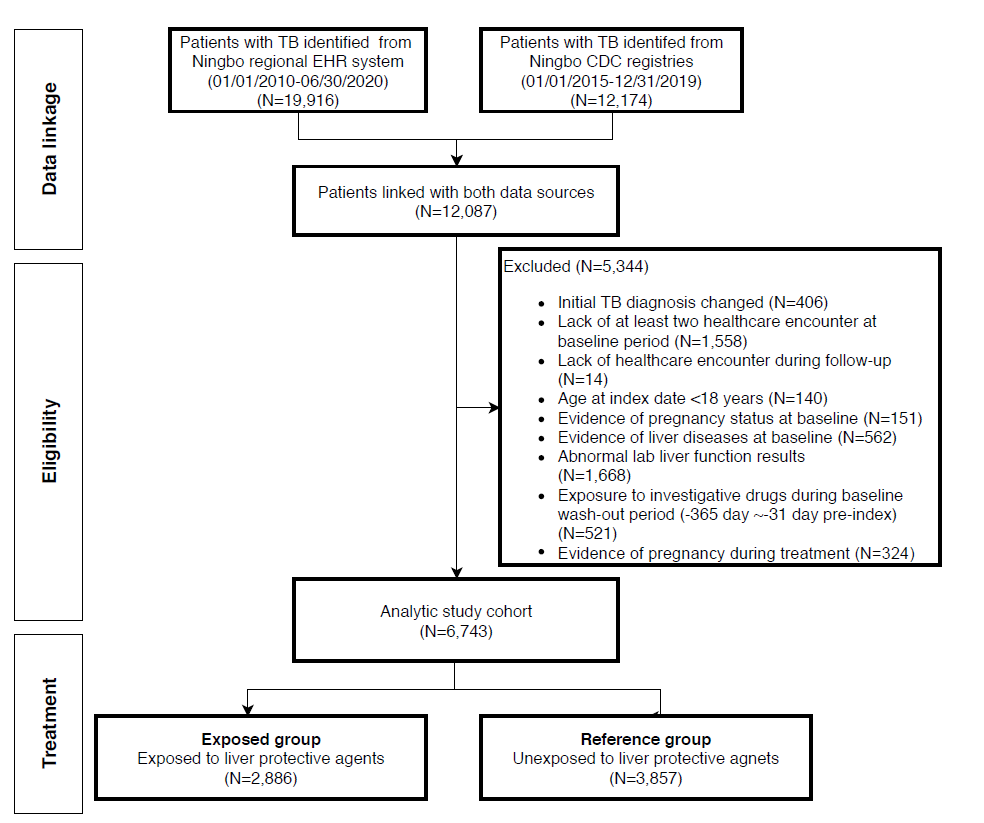


**Supplemental Figure 2**: Schematic overview of study design


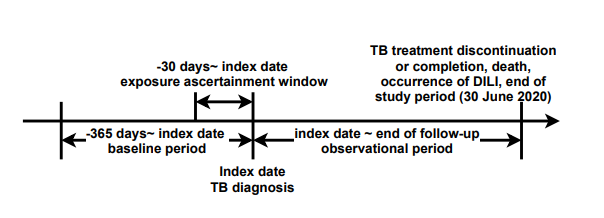

Supplement: Supplementary file 1 [file DataSheet1.docx]
